# BoLA-DRB3 Polymorphism is Associated with Differential Susceptibility to Bovine Leukemia Virus-Induced Lymphoma and Proviral Load

**DOI:** 10.3390/v12030352

**Published:** 2020-03-22

**Authors:** Chieh-Wen Lo, Liushiqi Borjigin, Susumu Saito, Koya Fukunaga, Etsuko Saitou, Katsunori Okazaki, Tetsuya Mizutani, Satoshi Wada, Shin-nosuke Takeshima, Yoko Aida

**Affiliations:** 1 Laboratory of Global Animal Resource Science, Graduate School of Agricultural and Life Sciences, the University of Tokyo, 2-1 Hirosawa, Wako, Saitama 351-0198, Japan; Chieh-Wen.lo@riken.jp; 2 Viral Infectious Diseases Unit, RIKEN, 2-1 Hirosawa, Wako, Saitama 351-0198, Japan; liushiqi@riken.jp (L.B.); susumu.saito@riken.jp (S.S.); takesima@jumonji-u.ac.jp (S.-n.T.); 3 Photonics Control Technology Team, RIKEN Center for Advanced Photonics, 2-1 Hirosawa, Wako, Saitama 351-0198, Japan; swada@riken.jp; 4 Nakamura Laboratory, Baton Zone Program, RIKEN Cluster for Science, Technology and Innovation Hub, 2-1 Hirosawa, Wako, Saitama 351-0198, Japan; 5 Research and Education Center for Prevention of Global Infectious Diseases of Animals, Tokyo University of Agriculture and Technology, 3-5-8 Saiwai-cho, Fuchu, Tokyo 183-8509, Japan; tmizutan20002000@gmail.com; 6 Laboratory for Pharmacogenomics, RIKEN Center for Integrative Medical Sciences, 1-7-22 Suehiro-cho, Tsurumi-ku, Yokohama City, Kanagawa 230-0045, Japan; koya.fukunaga@riken.jp; 7 Hyogo Prefectural Awaji Meat Inspection Center, 49-18 Shitoorinagata, Minamiawaji, Hyogo 656-0152, Japan; Etsuko_Saitou@pref.hyogo.lg.jp; 8 Laboratory of Microbiology and Immunology, Faculty of Pharmaceutical Sciences, Health Sciences University of Hokkaido, 1757 Kanazawa, Ishikari-Tobetsu, Hokkaido 061-0293, Japan; kokazaki@hoku-iryo-u.ac.jp; 9 Department of Food and Nutrition, Jumonji University, Niiza, Saitama 352-8510, Japan

**Keywords:** bovine leukemia virus, lymphoma, proviral load, *BoLA-DRB3*, polymorphism, susceptibility, resistance, association study

## Abstract

Bovine leukemia virus (BLV) is the causative agent of enzootic bovine leucosis. However, less than 5% of BLV-infected cattle will develop lymphoma, suggesting that, in addition to viral infection, host genetic polymorphisms might play a role in disease susceptibility. Bovine leukocyte antigen (BoLA)*-DRB3* is a highly polymorphic gene associated with BLV proviral load (PVL) susceptibility. Due to the fact that PVL is positively associated with disease progression, it is believed that controlling PVL can prevent lymphoma development. Thus, many studies have focused on the relationship between PVL and *BoLA-DRB3*. Despite this, there is little information regarding the relationship between lymphoma and *BoLA-DRB3*. Furthermore, whether or not PVL-associated *BoLA-DRB3* is linked to lymphoma-associated *BoLA-DRB3* has not been clarified. Here, we investigated whether or not lymphoma-associated *BoLA-DRB3* is correlated with PVL-associated *BoLA-DRB3*. We demonstrate that two *BoLA-DRB3* alleles were specifically associated with lymphoma resistance (*010:01 and *011:01), but no lymphoma-specific susceptibility alleles were found; furthermore, two other alleles, *002:01 and *012:01, were associated with PVL resistance and susceptibility, respectively. In contrast, lymphoma and PVL shared two resistance-associated (DRB3*014:01:01 and *009:02) *BoLA-DRB3* alleles. Interestingly, we found that PVL associated alleles, but not lymphoma associated alleles, are related with the anti-BLV gp51 antibody production level in cows. Overall, our study is the first to demonstrate that the *BoLA-DRB3* polymorphism confers differential susceptibility to BLV-induced lymphoma and PVL.

## 1. Introduction

Viral load in chronic infections with viruses, such as hepatitis B virus (HBV), hepatitis C virus (HCV), human T cell leukemia virus type 1 (HTLV-1), and human immunodeficiency virus type 1 (HIV-1), has been reported to determine the likelihood of pathogenesis and disease progression [1,2,3,4]. For retroviruses, whose genome integrates with the host genome, proviral load (PVL) is an important risk factor of viruss-associated disease prediction [5,6]. Bovine leukemia virus (BLV) is closely related to HTLV-1 and is the causative agent of enzootic bovine leukosis (EBL), a disease that is characterized by long-term symptoms, including persistent lymphocytosis (PL), which may culminate in B-cell lymphosarcoma [7,8]. Several studies indicate that BLV PVL is associated with BLV-related disease progression [9,10,11,12,13]. However, only 5% of infected cattle progress to develop lymphoma, suggesting that in addition to viral infection, host genetic polymorphisms might play a role in disease susceptibility.

The major histocompatibility complex (MHC), a highly polymorphic gene set, plays a crucial role in antigen presentation and immune responsiveness [14,15,16], and thus, it is associated with numerous infectious diseases. In cattle, the MHC system is known as bovine leukocyte antigen (BoLA). Several studies have identified genetic variations in *BoLA-DRB3*, a functionally important locus and the most highly polymorphic BoLA class II locus in cattle. To date, 330 *DRB3* alleles have been registered in the Immuno Polymorphism Database (IPD)- MHC database (https://www.ebi.ac.uk/ipd/mhc/group/BoLA/). The *BoLA-DRB3* polymorphism influences susceptibility to BLV-induced lymphoma [17,18,19], and to PVL [20,21,22]. As PVL is positively related to lymphoma development, it is possible that lymphoma-associated *BoLA-DRB3* is consistent with PVL-associated *BoLA-DRB3*. However, the consistency of the above association has not been studied yet.

Indeed, lymphoma development and viral replication depend on different cellular mechanisms, potentially leading to the differential susceptibility of lymphoma and PVL to *BoLA-DRB3*. It has been reported that proviral integration and BLV proteins are required for initial cell transformation [7,23]. However, the host immune system can remove transformed cells by lymphocyte activation via MHC molecules [24]. Due to the fact that MHC class II alleles affect antigen presentation and MHC expression levels in cancer cells [25,26], it is reasonable to hypothesize that MHC class II alleles would bind to peptides derived from viral or tumor antigens, and that the resulting complex would be recognized by CD4+ T cells. Consequently, some *BoLA-DRB3* alleles might specifically bind with the processed viral antigen, while others might specifically recognize the tumor antigens. Thus, it is likely that different *BoLA-DRB3* alleles are specifically associated with BLV-induced lymphoma and PVL. Consistent with this, PVL does not always correlate with lymphoma development, as many infected cows with a high PVL do not develop lymphoma. On the contrary, attenuated BLV-infected sheep were found to exhibit significantly lower PVL, but still developed lymphoma [27], suggesting that lymphoma and PVL may induce different susceptibilities, depending on different *BoLA-DRB3* polymorphisms. In this study, using asymptomatic and lymphoma Holstein cows randomly collected in a nationwide survey in Japan, we demonstrated that *BoLA-DRB3* polymorphism is associated with differential susceptibility to BLV-induced lymphoma and PVL.

## 2. Materials and Methods

### 2.1. Sample Collection and Diagnosis

Blood samples from 611 BLV-infected but clinically normal Holstein cows (asymptomatic cows; information summarized in Tables S1 and S6) and 221 BLV-infected Holstein cows with lymphoma (lymphoma cows; information summarized in Table S2) were randomly collected in a nationwide survey across Japan (32 prefectures out of 47), and the genomic DNA and plasma from peripheral blood were isolated. The subclinical stage of BLV infection was diagnosed according to the lymphocyte count (cells/μL) and the age of each cow (≤ 8,500 = normal and ≥ 13,000 = lymphocytosis for cows aged 2–3 years; ≤ 5,500 = normal and ≥ 7,500 = lymphocytosis for cows aged ≥ 6 years). Asymptomatic cows were defined as BLV-infected but clinically and hematologically normal cattle; PL cows were defined as BLV-infected but clinically normal cattle showing with an increase in the number of apparently normal B lymphocytes. Subsequently, lymphoma was diagnosed by both gross and histological observation and by detecting atypical mononuclear cells in the slaughterhouse. In this study, PL cases were excluded and used only samples from asymptomatic cows and lymphoma cows.

### 2.2. BLV Proviral Load Determination

BLV infection was estimated by BLV-CoCoMo-qPCR-2 (RIKEN Genesis, Kanagawa, Japan), as previously described [9,10,28,29,30]. Briefly, the BLV-LTR region was amplified in a reaction mixture containing THUNDERBIRD Probe qPCR Mix (Toyobo, Tokyo, Japan), CoCoMo FRW primer, CoCoMo REV primer, FAM-BLV probe, and 150 ng of template DNA. In addition, the *BoLA-DRA* region was amplified as internal control. The proviral load was calculated using the following equation: (number of BLV-LTR copies /number of *BoLA-DRA* copies) × 10^5^ cells.

### 2.3. BoLA-DRB3 Genotyping

*BoLA-DRB3* alleles were determined using the PCR-sequenced-based typing (SBT) method, as previously described [31]. Briefly, *BoLA-DRB3* exon 2 was amplified by single-step PCR using the DRB3 forward (5’-CGCTCCTGTGAYCAGATCTATCC-3’) and DRB3 reverse (5’-CACCCCCGCGCTCACC-3’) primer set. The PCR products were purified by the ExoSAP-IT PCR product purification kit (USB Corp., Cleveland, OH, USA), and then sequenced using the ABI PRISM BigDye1.1 Terminator Cycle Sequencing Ready Reaction Kit (Applied Biosystems, Foster City, CA, USA). The sequence data were then analyzed using Assign 400ATF ver. 1.0.2.41 software (Gonexio Genomics, Fremantle, Australia) to determine the *BoLA-DRB3* genotype.

### 2.4. Detection of Anti-BLV gp51 Antibody by Enzyme-Linked Immunosorbent Assay (ELISA)

The anti-BLV gp51 antibody was measured with an anti-BLV antibody ELISA Kit (JNC, Tokyo, Japan), according to the manufacturer’s instructions. Two-fold serial dilutions of plasma samples starting at 1:16 were tested by the ELISA Kit. The OD value in each DRB3 group was compared at each dilution.

### 2.5. Association Study and Statistical Analysis

An association study based on Fisher’s exact test was performed by comparing the allele and genotype frequencies between asymptomatic and lymphoma cows or low PVL and high PVL cows. The results were penalized with the Benjamini–Hochberg (BH) procedure to correct for the false positive rate. Each allele or genotype was ranked based on their *p*-value starting from the smallest one. The BH value was calculated based on the equation *p*-value rank / total allele (genotype) number × 0.05). The alleles or genotypes with *p*-value < BH value and odds ratio (OR) < 1 were categorized as resistance alleles. In contrast, those with *p*-value < BH value and OR > 1 were defined as susceptibility alleles or genotypes. The association of cow mean age and birth location with lymphoma or PVL was evaluated by the Mann–Whitney U test and Tukey’s multiple comparison test, respectively. When we confirmed the association between age and lymphoma or PVL, we performed logistic regression analyses to adjust for age. To evaluate a multiplicative interaction between *BoLA-DRB3* alleles, we introduced the interaction term in a logistic regression model as conditional analysis [32]. We assessed the significance level of the association study by applying a Bonferroni correction according to the number of assessed alleles (adjusted *p* < 0.05). All calculations were performed using R software (version 3.5.0, R Foundation for Statistical Computing, Vienna, Austria).

## 3. Results

### 3.1. PVL Is Not Fully Correlated with Lymphoma Development

**Figure 1:**
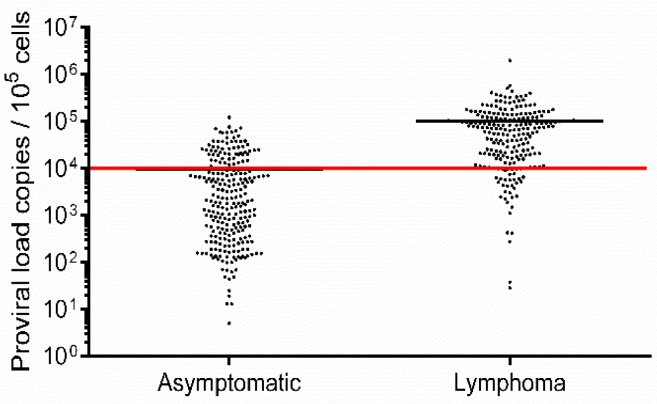
Proviral load (PVL) estimation in Bovine leukemia virus (BLV)-infected but clinically and hematologically normal cows (asymptomatic cows) and BLV-infected cows with lymphoma (lymphoma cows). Blood samples were obtained from 250 asymptomatic (Table S1) and 221 lymphoma (Table S2) cows in a nationwide survey in Japan. BLV infection was analyzed using BLV-CoCoMo-qPCR-2. The red line represents a BLV PVL of 10,000 copies/10^5^ cell, which was set as the threshold between high- and low-PVL cows.

The PVL of 250 asymptomatic cows (Table S1) ranged from 5–120,482 copies/10^5^ cells (mean: 9401 copies/10^5^ cells), while in 221 lymphoma cows (Table S2), the PVL ranged from 28–1,960,674 copies/10^5^ cells (mean 99,522 copies/10^5^ cells; Figure 1). This difference suggested that animals with a high BLV PVL were at a higher risk of developing lymphoma. Our previous report indicated that cows with a PVL of greater than 14,000 copies/10^5^ cells secreted BLV into nasal mucus [30], and BLV provirus was detected in milk samples from cows when the PVL in blood samples was higher than 10,000 copies/10^5^ cells [33]. These results suggest that a PVL around 10,000 copies/10^5^ cells in blood might be an indicator of efficient BLV spreading within the whole body and thus this is a relatively high number. Therefore, a BLV PVL of 10,000 copies/10^5^ cells was set as a threshold to distinguish between high-PVL (HPVL) and low-PVL (LPVL) cows (Figure 1), which is also in line with our previous study [19]. Consistently, in lymphoma and asymptomatic cows, the mean PVL was found to be above and below this threshold, respectively (Figure 1). However, 62 HPVL cows remained asymptomatic, whereas 37 LPVL cows developed lymphoma (Table 1), indicating that lymphoma development is not fully correlated with the PVL. This could be because BLV-induced lymphoma and BLV PVLs are associated with different *BoLA-DRB3* alleles.

### 3.2. Association Study of BoLA-DRB3 with Lymphoma

**Figure 2:**
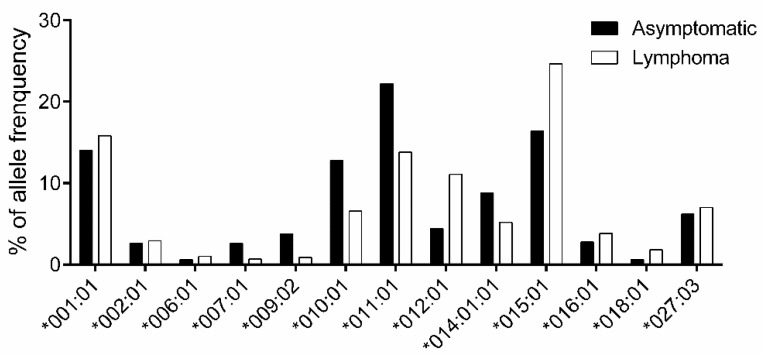
Comparison of *BoLA-DRB3* allele frequencies between asymptomatic and lymphoma cows. Allele frequency in 250 asymptomatic (■) and 221 lymphoma (□) cows were calculated for each *BoLA-DRB3* allele (Table S3); 13 out of 32 alleles with frequency > 1% are shown. The X-axis shows the allele name and the Y-axis shows allele frequency (%) for each *BoLA-DRB3* allele.

Next, to explore the association between *BoLA-DRB3* and lymphoma, all 250 asymptomatic and 221 lymphoma cows were typed for *BoLA-DRB3* alleles (Table S3). The alleles with frequencies > 1% are shown in Figure 2. An association study based on Fisher’s exact test found that DRB3*009:02 (OR = 0.23), DRB3*010:01 (OR = 0.48), DRB3*011:01 (OR = 0.56), and DRB3*014:01:01 (OR = 0.57) were classified as lymphoma resistance alleles, whereas DRB3*012:01 (OR = 2.71) and DRB3*015:01 (OR = 1.67) were identified as lymphoma susceptibility alleles (Table 2). 

To exclude the effect from other potential factors that might associate with lymphoma development such as cow origin and age, we then applied multivariable logistic regression to adjust for the effect of these potential factors. Our calculation indicates that age showed a significant association with disease susceptibility (*p* = 6.56 × 10^−6^). However, no significant difference was observed between location and lymphoma susceptibility (*p* = 0.182). Therefore, we performed the logistic regression analysis adjusted by age in the only association study of lymphoma. After studying the association between each *BoLA-DRB3* allele and lymphoma susceptibility, we conducted a stepwise conditional analysis with respect to the top-associated *BoLA-DRB3* alleles (Table S4). In Table 3, a conditional analysis of DRB3*011:01 revealed an independent association with DRB3*009:02 and DRB3*010:01. A subsequent conditional analysis regarding DRB3*009:02 and DRB3*011:01 revealed an independent association with DRB3*010:01 and DRB3*014:01:01. Next, the subsequent conditional analysis of DRB3*009:02, DRB3*010:01, and DRB3*011:01 revealed an independent association with DRB3*014:01:01. After conditioning DRB3*009:02, DRB3*010:01, DRB3*011:01, and DRB3*014:01:01, no significant association locus was observed. We then conducted a multivariate regression analysis incorporating the four associated *BoLA-DRB3* alleles (DRB3*009:02, DRB3*010:01, DRB3*011:01, and DRB3*014:01:01). We identified that all were independently associated with lymphoma resistance (Table 3).

**Figure 3:**
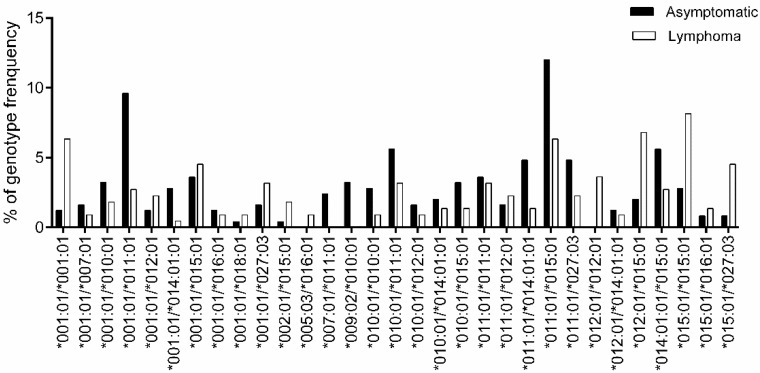
Comparison of *BoLA-DRB3* genotype frequency between asymptomatic and lymphoma cows. Genotype frequency of 250 asymptomatic (■) and 221 lymphoma (□) cows was calculated for each *BoLA-DRB3* genotype (Table S4); a total of 31 out of 94 genotypes with frequency > 1% are shown. The X-axis and Y-axis show the genotype name and frequency (%) for each *BoLA-DRB3* genotype, respectively.

For the genotype association study, genotypes with frequencies > 1% are shown in Figure 3 (complete genotype frequencies are summarized in Table S5). However, no genotypes reached statistical significance in terms of their association with lymphoma development after BH correction to adjust the false discovery rate (Table 4).

### 3.3. Association Study of BoLA-DRB3 with PVL

**Figure 4:**
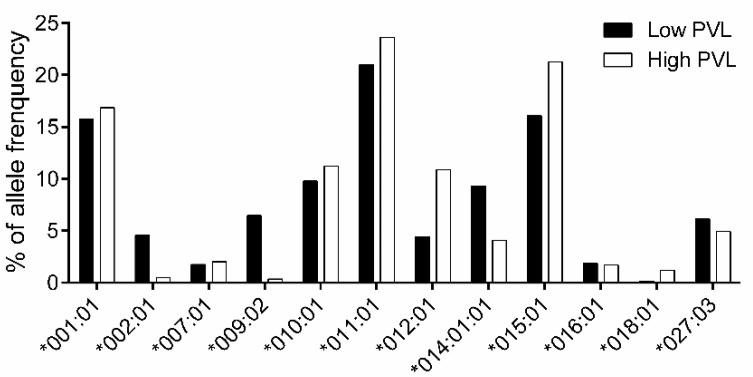
Comparison of *BoLA-DRB3* allele frequencies between LPVL and HPVL cows. *BoLA-DRB3* allele frequencies in LPVL and HPVL cows. The 611 asymptomatic cows comprised 317 LPVL and 294 HPVL individuals. The allele frequencies were calculated in LPVL (■) and HPVL cows (□) for each *BoLA-DRB3* allele (Table S6). Total of 12 out of 26 alleles with frequency > 1% are shown.

Subsequently, to determine the association between *BoLA-DRB3* and BLV PVL, we selected an additional 361 asymptomatic cows, in addition to the original 250 asymptomatic cows (used in Figure 1, Figure 2 and Figure 3). A total of 611 asymptomatic cows were then divided into the HPVL group (*n* = 294; Table S6) and LPVL group (*n* = 317; Table S6). The frequencies of *BoLA-DRB3* alleles from LPVL cows and HPVL cows were calculated by Fisher’s exact test, and *p*-values and ORs were estimated for each allele (Table S7). The analysis of allele frequencies (Figure 4) and association (Table 5) established DRB3*002:01 (OR = 0.15), *009:02 (OR = 0.07), and *014:01:01 (OR = 0.61) as BLV PVL resistance alleles, consistent with previous findings [20]. In addition, DRB3*012:01 (OR = 3.84) was identified as a susceptibility allele. 

Next, to exclude the bias that might occur in Fisher’s exact test, we assessed the association between PVL and other potential factors, including age and the cows’ birth location. However, no association between age (*p* = 0.170)/location (*p* = 0.991) and PVL was observed. After studying the association between each *BoLA-DRB3* allele and PVL, we conducted stepwise conditional analysis, with respect to the top-associated *BoLA-DRB3* alleles (Table S8). In Table 6, a conditional analysis of DRB3*009:02 revealed an independent association with DRB3*002:01, DRB3*012:01, and DRB3*014:01:01. A subsequent conditional analysis regarding DRB3*009:02 and DRB3*014:01:01 revealed an independent association with DRB3*002:01 and DRB3*012:01. Next, a conditional analysis of DRB3*009:02, DRB3*012:01, and DRB3*014:01:01 revealed an independent association with DRB3*002:01. After conditioning DRB3*002:01, DRB3*009:02, DRB3*012:01, and DRB3*014:01:01, no significant association locus was observed. We then conducted a multivariate regression analysis, incorporating the four associated *BoLA-DRB3* alleles (DRB3*002:01, DRB3*009:02, DRB3*012:01, and DRB3*014:01:01). We identified that DRB3*002:01, DRB3*009:02, and DRB3*014:01:01 are resistance alleles and DRB3*012:01 is a susceptibility allele independently associated with PVL (Table 6).

**Figure 5:**
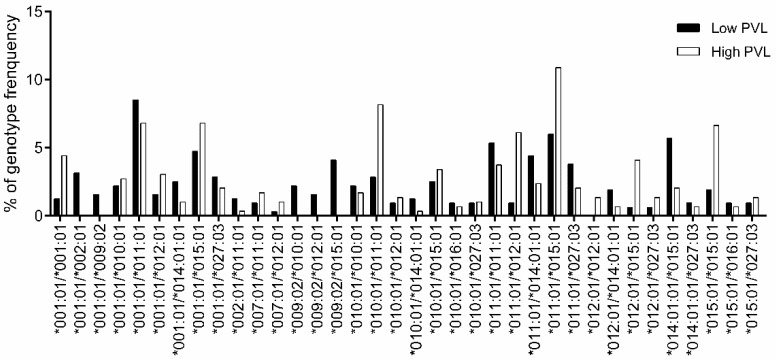
Comparison of *BoLA-DRB3* genotype frequencies between HPVL and LPVL cows. The genotype frequencies of 317 LPVL (■) and 294 HPVL cows (□) were calculated for each *BoLA-DRB3* genotype (Table S9); a total of 36 out of 92 genotypes with frequencies > 1% are shown. The X-axis and Y-axis show the genotype name and frequency (%) for each *BoLA-DRB3* genotype, respectively.

For genotype association study (Table S9), the genotypes with frequency > 1% are shown in Figure 5. The Fisher’s exact test of genotype association (Table 7) indicated that DRB3*009:02/*015:01 (OR = 0) was determined as the resistance genotype. In contrast, DRB3*011:01/*012:01 (OR = 6.83), was determined as the susceptibility genotype.

### 3.4. Differential Susceptibility of BoLA-DRB3 Polymorphisms to Lymphoma and PVL

**Figure 6:**
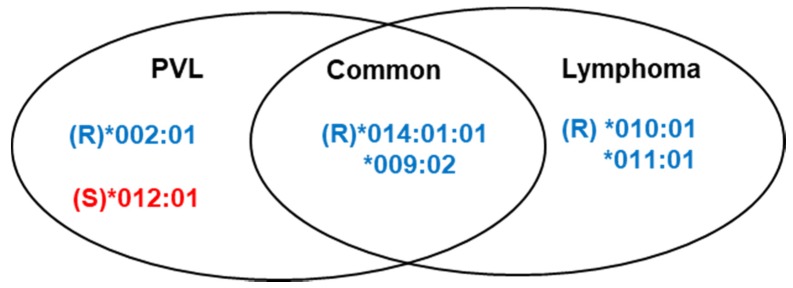
Summary of the differences in *BoLA-DRB3* allele-associated proviral load (PVL) and lymphoma susceptibility, based on the logistic regression association study results. R, resistance; S, susceptibility.

We compared the effect of *BoLA-DRB3* on cow susceptibility to lymphoma and PVL, based on the multivariable logistic regression analysis in Figure 2 and Figure 4. Several different types of *BoLA-DRB3* alleles were found to be associated with BLV-induced lymphoma and BLV PVL (Figure 6). There were two lymphoma resistance alleles, DRB3*010:01, and DRB3*011:01, but no susceptibility alleles were identified. In addition, one allele associated with PVL resistance, DRB3*002:01, and one PVL susceptibility allele, DRB3*012:01, were found. Two resistance alleles, DRB3*009:02 and DRB3*014:01:01, were commonly identified in lymphoma and BLV PVL.

### 3.5. BoLA-DRB3 Polymorphisms Are Associated with anti-BLV Antibody Production Levels

**Figure 7:**
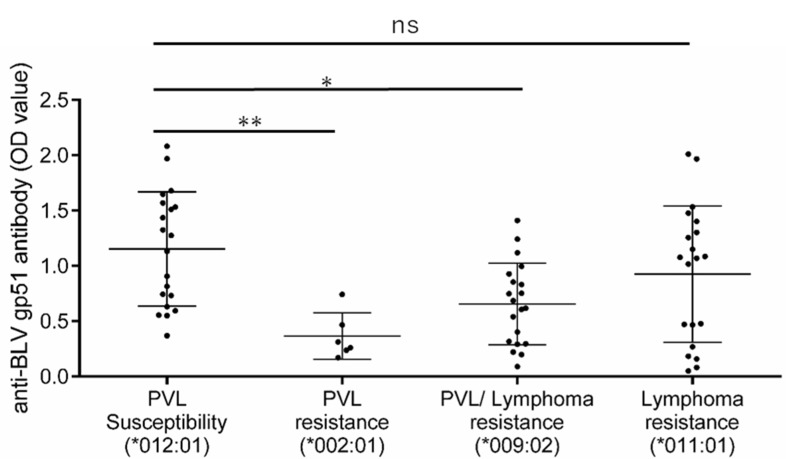
Differential anti-BLV antibody production level in cows with proviral load (PVL) susceptibility, PVL resistance, PVL/lymphoma resistance, and lymphoma-resistance *BoLA-DRB3* allele. The PVL susceptibility group consisted of cows with the PVL susceptibility allele DRB3*012:01 (*n* = 20). The PVL resistance group consisted of cows with the PVL resistance allele DRB3*002:01 (*n* = 6). The PVL/lymphoma resistance group consisted of cows with the PVL resistance allele DRB3*009:02 (*n* = 20). The lymphoma resistance group consisted of the lymphoma resistance allele *011:01 (*n* = 20). The anti-BLV gp51 antibody was measured in plasma at a 2^11^ dilution level. Optical Density (OD) value data represent the mean ± SD. Statistical comparisons were performed by one-way ANOVA. *, *p* < 0.05. **, *p* < 0.01. ns, not significant.

Finally, we tried to link the potential biological functions with *BoLA-DRB3* polymorphisms. Previously, it has been demonstrated that PVL resistance and susceptibility alleles are associated with anti-BLV antibody production levels [34]. Here, we hypothesized that only PVL-associated alleles, but not lymphoma-associated alleles, would be related to viral antigen-induced immune responses. To test this, we compared the anti-BLV antibody (anti-gp51) production level between the cows with the PVL susceptibility allele (DRB3*012:01), PVL resistance allele (DRB3*002:01), PVL/lymphoma resistance allele (DRB3*009:02), and lymphoma-specific resistance allele (DRB3*011:01), as shown in the summary in Figure 6. Two-fold serially-diluted plasma samples were tested by ELISA and the OD value in each *BoLA-DRB3* group was compared at the dilution of 1:2048. Cows with the PVL resistance allele (PVL resistance group and PVL/lymphoma resistance group) had significantly lower anti-gp51 production levels compared to those in cows carrying the PVL susceptibility allele (*p* = 0.006 and *p* = 0.012 respectively; Figure 7). However, cows with the lymphoma specific resistance allele did not have explicitly and significantly different levels of anti-gp51, compared to those in animals with the PVL susceptibility allele (*p* = 0.474), suggesting that the lymphoma specific associated allele has a lesser effect on anti-gp51 production than the PVL associated allele.

## 4. Discussion

In the present study, by using both Fisher’s exact test and multivariable logistic regression analysis, we showed for the first time that the susceptibility to BLV-induced lymphoma and PVL is affected by different *BoLA-DRB3* polymorphisms. For example, two *BoLA-DRB3* alleles, DRB3*010:01, and DRB3*011:01, were found to be associated with resistance to lymphoma but not to PVL. In addition, DRB3*002:01 was specifically associated with PVL resistance. In contrast, we found that DRB3*009:02 was common between lymphoma and PVL resistance, in line with the reciprocal association between PVL and lymphoma development. Thus, we might conclude that host polymorphisms at the *BoLA-DRB3* locus are an important factor in both PVL and lymphoma development; interestingly, the PVL-associated *BoLA-DRB3* allele did not show a major correlation with the lymphoma-associated *BoLA-DRB3* allele. The potential reason for the differential susceptibility to PVL and Lymphoma might be the differential immune response, which depends on *BoLA-DRB3* polymorphisms, as we found that the level of anti-BLV antibody is related to the PVL-associated allele but not the lymphoma-associated allele, suggesting that lymphoma specific-associated alleles have a lesser effect on anti-BLV antibody production than PVL-associated alleles.

Some discrepancies were observed between the association based on Fisher’s exact test and multivariable logistic regression analysis. For example, DRB3*015:01 was indicated as a lymphoma susceptibility allele by Fisher’s exact test, but not by multivariable logistic regression analysis. The inconsistencies can also be found in DRB3*012:01 and DRB3*014:01:01. This is a common problem between these two statistical methods, as Fisher’s exact test includes all factors, such as environmental and genetically factors, that together influence *BoLA-DRB3* polymorphisms. In contrast, multivariable logistic regression can adjust for the effect of other associated factors, such as age, for the lymphoma association study. Therefore, further experiments for allele functional confirmation are needed.

It is not clear how most BLV-infected cattle do not develop bovine leukosis. The following findings in our study may help solve this issue: (i) In asymptomatic cows, two of the major *BoLA-DRB3* alleles, DRB3*011:01 (22%) and DRB3*010:01 (12%), were significantly associated with lymphoma resistance, but were unrelated to PVL. This may explain why some HPVL cows remained asymptomatic. (ii) Susceptibility alleles specifically associated with lymphoma were absent in Japanese Holstein cows, suggesting that malignant transformation requires other factors, besides *BoLA-DRB3* polymorphism. For instance, the deregulation of lymphocyte homeostasis is known to lead to leukemia [35]. Provirus integration close to cancer-driver sites and transcriptionally active regions may affect host gene expression [23,36,37]. The viral accessory proteins Tax and G4 also play a crucial role in cell transformation [38]. Besides, p53 mutation [39,40] and tumor necrosis factor-α polymorphisms [41] are also related to lymphoma development.

The major function of MHC class II molecules is to present antigens for T cells to activate the adaptive immune response. It is possible that PVL-specific *BoLA-DRB3* and lymphoma-specific *BoLA-DRB3* recognize different antigens and thus the subsequent immune response targeting the virus or tumor cells, respectively. To link *BoLA-DRB3* polymorphisms with their biological functions, we tested the anti-BLV gp51 antibody level in cows with the PVL-associated *BoLA-DRB3* allele and lymphoma-associated *BoLA-DRB3* allele. Interestingly, significantly different anti-gp51 levels were found between cows with PVL resistance and PVL susceptibility alleles. However, no significant difference was found in cows carrying lymphoma-specific resistance alleles compared to those in cows carrying PVL susceptibility alleles. This result is in line with the hypothesis that proteins encoded by PVL-associated and lymphoma-associated *BoLA-DRB3* alleles recognize different antigens and thus trigger different subsequent immune responses. Similar to that in a previous study, we found that cows with PVL resistance alleles exhibit significantly lower anti-gp51 levels than cows with PVL susceptibility alleles [34]. This is probably because we measured anti-gp51 levels in steady state virus infections, and thus, the antibody concentration might change and correlate with the viral titer in cows. As a result, in PVL resistance cows, which are associated with low viral expression levels, a low anti-BLV antibody level would be detected. In addition to humoral immunity, whether *BoLA-DRB3* polymorphisms are associated with effects on cytotoxic T lymphocytes needs further study.

In the PVL association study, DRB3*015:01 has been reported as a PVL susceptibility allele in our and other studies [19,34]. However, we found only one PVL susceptibility allele, DRB3*012:01, in the current investigation. This difference might be due to the sample collection bias and also the statistical analysis method. For the lymphoma association study, it has been reported that the BoLA-DRB3*018:02, DRB3*032:02, and DRB3*009:01 alleles are associated with the susceptibility to BLV-induced lymphoma, whereas DRB3*001:01 and DRB3*011:01 are involved in lymphoma resistance in Iranian Holstein cows [17]. In the present study, DRB3*011:01 was confirmed to be a resistance allele. The other identified alleles were different from those previously reported, suggesting that regional genetic variations may exist. Indeed, ethnicity-related differences in the frequency of human MHC alleles have been observed [42,43]. Furthermore, allelic diversity in the *BoLA* locus between cattle breeds has been previously demonstrated [44,45,46,47]. This variability is strongly influenced by selective pressures such as exposure to infectious diseases and breed origin. Therefore, the association between the *BoLA-DRB3* locus and the resistance or susceptibility to BLV-induced lymphoma, as well as the regulation of PVL, should be further explored in different countries and in distinct cow breeds.

In conclusion, we have demonstrated for the first time that BLV-induced lymphoma and PVL are associated with different *BoLA-DRB3* alleles in Holstein cows in Japan. Although BLV infects cattle worldwide, effective treatments and vaccines are not available. Consequently, breed selection based on *BoLA-DRB3* polymorphism is a promising strategy to reduce the burden of BLV-induced lymphoma. Contrarily, the sporadic inconsistency between PVL and terminal diseases might be a common phenomenon, due to host genetic polymorphisms during different infectious viral diseases. Indeed, partial inconsistency between PVL and the related symptoms was also observed in HTLV-1-infected patients [48]. As BLV is closely related to HTLV-1, the consistency between the susceptibility of host genetic polymorphisms with PVL and HTLV-1-related symptoms is worth confirming.

## Figures and Tables

**Table 1:** Summary of PVL distribution in asymptomatic cows and lymphoma cows.

Status	Asymptomatic (*n* = 250)	Lymphoma (*n* = 221)
Low proviral load ^1^	188	37
High proviral load ^2^	62	184

^1^ A PVL of < 10^4^ copies/10^5^ cells was considered Low proviral load; ^2^ a PVL of > 10^4^ copies/10^5^ cells was considered High proviral load.

**Table 2:** Fisher’s exact test based association analysis of *BoLA-DRB3* alleles in asymptomatic and lymphoma cows.

*BoLA-DRB3* Allele	Asymptomatic (250 Cattle)	Lymphoma (221 Cattle)	OR	*p*-Value	*p*-Value Rank (I)	BH Value (I/Allele Number)*0.05	Susceptibility
*001:01	70	70	1.156	0.463			-
*002:01	13	13	1.135	1.000			-
*006:01	3	3	1.132	1.000			-
*007:01	13	3	0.256	0.024	6	0.009	-
*009:02	19	4	0.231	0.005	5	0.008	R
*010:01	64	29	0.478	0.001	2	0.003	R
*011:01	111	61	0.561	0.001	2	0.003	R
*012:01	22	49	2.709	0.000	1	0.002	S
*014:01:01	44	23	0.569	0.042			-
*015:01	82	109	1.669	0.002	4	0.006	S
*016:01	14	17	1.389	0.578			-
*018:01	3	8	3.054	0.127			-
*027:03	31	31	1.141	0.693			-

The Benjamini–Hochberg (BH) procedure was performed to adjust the false positive rate. Alleles with a *p*-value < BH value were defined as susceptibility (S) with an odds ratio (OR) > 1 and as resistance (R) with an OR < 1. BH value = (*p*-value rank/total allele number) × 0.05.

**Table 3:** Logistic regression analysis-based association study of *BoLA-DRB3* alleles in asymptomatic and lymphoma cows after adjustments for age.

*BoLA-DRB3* Allele	Univariate				Multivariate			
	*p*-Value	OR	L95	U95	*p*-Value	OR	L95 ^1^	U95 ^2^
*009:02	0.002	0.10	0.02	0.43	4.27 × 10^−4^	0.07	0.01	0.30
*010:01	0.008	0.52	0.32	0.84	7.38 × 10^−4^	0.43	0.026	0.70
*011:01	9.91 × 10^−4^	0.53	0.36	0.77	5.77 × 10^−6^	0.40	0.27	0.59
*014:01:01	0.026	0.53	0.30	0.93	7.82 × 10^−4^	0.36	0.20	0.66

^1^ L95, lower 95% confidence interval. ^2^ U95, upper 95% confidence interval.

**Table 4:** Fisher’s exact test based association analysis of *BoLA-DRB3* genotypes in asymptomatic and lymphoma cows.

*BoLA-DRB3* Genotype	Asymptomatic (212 Cattle)	Lymphoma (221 Cattle)	OR	*p*-Value	*p*-Value Rank (I)	BH Value (I/Genotype Number)*0.05	Susceptibility
*001:01/*001:01	3	14	5.568	0.005			-
*001:01/*007:01	4	2	0.562	0.689			-
*001:01/*010:01	8	4	0.558	0.393			-
*001:01/*011:01	24	6	0.263	0.002	1	0.0005	-
*001:01/*012:01	3	5	1.906	0.483			-
*001:01/*014:01:01	7	1	0.158	0.393			-
*001:01/*015:01	9	10	1.269	0.645			-
*001:01/* 016:01	3	2	0.752	1.000			-
*001:01/*018:01	1	2	2.274	0.603			-
*001:01/*027:03	4	7	2.012	0.362			-
*002:01/*015:01	1	4	4.590	0.191			-
*005:03/*016:01	0	2	-	0.220			-
*007:01/*011:01	6	0	0	0.057			-
*009:02/*010:01	8	0	-	0.008			-
*010:01/*010:01	7	2	0.317	0.183			-
*010:01/*011:01	14	7	0.551	0.264			-
*010:01/*012:01	4	2	0.562	0.689			-
*010:01/*014:01:01	5	3	0.674	0.728			-
*010:01/*015:01	8	3	0.416	0.231			-
*011:01/*011:01	9	7	0.876	1.000			-
*011:01/*012:01	4	5	1.424	0.740			-
*011:01/*014:01:01	12	3	0.273	0.037			-
*011:01/*015:01	30	14	0.496	0.039			-
*011:01/*027:03	12	5	0.459	0.215			-
*012:01/*012:01	0	8	-	0.002	1	0.0005	-
*012:01/*014:01:01	3	2	0.752	1.000			-
*012:01/*015:01	5	15	3.568	0.011			-
*014:01:01/*015:01	4	1	0.280	0.377			-
*015:01/*015:01	7	18	3.078	0.013			-
*015:01/*016:01	2	3	1.706	0.669			-
*015:01/*027:03	2	10	5.877	0.016			-

The Benjamini–Hochberg (BH) procedure was performed, to adjust the false positive rate. Genotypes with a *p*-value < BH value were defined as susceptibility (S) with an odds ratio (OR) > 1 and as resistance (R) with an OR < 1. BH value = (*p*-value rank / total allele number) × 0.05.

**Table 5:** Fisher’s exact test based association analysis of *BoLA-DRB3* alleles in low PVL and high PVL cows.

*BoLA-DRB3* Allele	Low PVL (317 Cattle)	High PVL (294 Cattle)	OR	*p*-Value	*p*-Value Rank (I)	BH Value (I/Genotype Number)*0.05	Susceptibility
*001:01	100	99	1.8681	0.6046			-
*002:01	29	3	0.1458	< 0.0001	1	0.0019	R
*007:01	11	12	1.5744	0.8322			-
*009:02	41	2	0.0685	< 0.0001	1	0.0019	R
*010:01	62	66	1.7989	0.4262			-
*011:01	133	139	2.2967	0.1933			-
*012:01	28	64	3.8383	< 0.0001	1	0.0019	S
*014:01:01	59	24	0.6068	0.0004	4	0.0077	R
*015:01	102	125	2.5463	0.0224	5	0.0096	-
*016:01	12	10	1.1962	0.8314			-
*018:01	1	7	9.968	0.0319			-
*027:03	39	29	1.1249	0.369			-

Benjamini–Hochberg (BH) procedure was performed to adjust the false positive rate. Alleles with *p*-value < BH value were defined as susceptibility (S) with odds ratio (OR) > 1, and as resistance (R) with OR < 1. BH value = (*p*-value rank / total allele number) × 0.05.

**Table 6:** Logistic regression analysis based association study of *BoLA-DRB3* alleles in low PVL and high PVL cows after adjustment of age.

*BoLA-DRB3* Allele	Univariate				Multivariate			
	*p*-Value	OR	L95	U95	*p*-Value	OR	L95	U95
*002:01	6.28 × 10^−4^	0.13	0.04	0.41	5.19 × 10^−4^	0.12	0.04	0.40
*009:02	3.29 × 10^−5^	0.05	0.01	0.20	1.42 × 10^−5^	0.04	0.01	0.17
*012:01	4.00 × 10^−5^	2.65	1.66	4.22	3.20 × 10^−4^	2.51	1.52	4.15
*014:01:01	2.40 × 10^−4^	0.39	0.23	0.64	2.10 × 10^−5^	0.31	0.18	0.53

L95, lower 95% confidence interval. U95, upper 95% confidence interval.

**Table 7:** Fisher’s exact test based association analysis of *BoLA-DRB3* genotypes in low PVL and high PVL cows.

*BoLA-DRB3* Genotype	Low PVL (317 Cattle)	High PVL (294 Cattle)	OR	*p*-Value	*p*-Value Rank (I)	BH Value (I/Genotype Number)*0.05	Susceptibility
*001:01/*001:01	4	13	3.6201	0.0245			-
*001:01/*002:01	10	0	0.0000	0.0019	3	0.0016	-
*001:01/*009:02	5	0	0.0000	0.0624			-
*001:01/*010:01	7	8	1.2388	0.7958			-
*001:01/*011:01	27	20	0.7840	0.4511			-
*001:01/*012:01	5	9	1.9705	0.2824			-
*001:01/*014:01:01	8	3	0.3982	0.2260			-
*001:01/*015:01	15	20	1.4696	0.2990			-
*001:01/*027:03	9	6	0.7130	0.6067			-
*002:01/*011:01	4	1	0.2671	0.3747			-
*007:01/*011:01	3	5	1.8108	0.4908			-
*007:01/*012:01	1	3	3.2577	0.3559			-
*009:02/*010:01	7	0	0.0000	0.0156			-
*009:02/*012:01	5	0	0.0000	0.0624			-
*009:02/*015:01	13	0	0.0000	0.0002	1	0.0005	R
*010:01/*010:01	7	5	0.7662	0.7741			-
*010:01/*011:01	9	24	3.0420	0.0039			-
*010:01/*012:01	3	4	1.4437	0.7160			-
*010:01/*014:01:01	4	1	0.2671	0.3747			-
*010:01/*015:01	8	10	1.3600	0.6341			-
*010:01/*016:01	3	2	0.7169	1.0000			-
*010:01/*027:03	3	3	1.0790	1.0000			-
*011:01/*011:01	17	11	0.6859	0.4393			-
*011:01/*012:01	3	18	6.8261	0.0005	2	0.0011	S
*011:01/*014:01:01	14	7	0.5279	0.1883			-
*011:01/*015:01	19	32	1.9156	0.0396			-
*011:01/*027:03	12	6	0.5295	0.2370			-
*012:01/*012:01	0	4	-	0.0530			-
*012:01/*014:01:01	6	2	0.3550	0.2886			-
*012:01/*015:01	2	12	6.7021	0.0053			-
*012:01/*027:03	2	4	2.1724	0.4351			-
*014:01:01/*015:01	18	6	0.3461	0.0223			-
*014:01:01/*027:03	3	2	0.7169	1.0000			-
*015:01/*015:01	6	19	3.5812	0.0067			-
*015:01/*016:01	3	2	0.7169	1.0000			-
*015:01/*027:03	3	4	1.4437	0.7160			-

The Benjamini–Hochberg (BH) procedure was performed to adjust the false positive rate. Alleles with a *p*-value < BH value were defined as susceptibility (S) with an odds ratio (OR) > 1 and as resistance (R), with an OR < 1. BH value = (*p*-value rank / total allele number) × 0.05.

## Supplementary Materials

The following are available online at https://www.mdpi.com/1999-4915/12/3/352/s1, Table S1. Sample information of asymptomatic cows in *BoLA-DRB3* and BLV-induced lymphoma association study. Table S2. Sample information of lymphoma cows in *BoLA-DRB3* and BLV-induced lymphoma association study. Table S3. Association study of *BoLA-DRB3* alleles between asymptomatic cows and lymphoma cows. Table S4. Results of the stepwise conditional analysis of *BoLA-DRB3* alleles in asymptomatic and lymphoma cows. Table S5. Association study of *BoLA-DRB3* genotypes between Asymptomatic cows and lymphoma cows. Table S6. Sample information of LPVL and HPVL cows in PVL association study. Table S7. Association study of *BoLA-DRB3* alleles between LPVL and HPVL cows. Table S8. Results of the stepwise conditional analysis of *BoLA-DRB3* alleles in LPVL and HPVL cows. Table S9. Association study of *BoLA-DRB3* genotypes between LPVL and HPVL cows.
